# Severe corneal melting and perforation secondary to chronic dacryocystitis due to delayed ophthalmology consultation

**DOI:** 10.1093/omcr/omab009

**Published:** 2021-04-28

**Authors:** Daisuke Nagasato, Hitoshi Tabuchi, Tomofusa Yamauchi, Hitoshi Imamura, Yoshie Shimizu

**Affiliations:** 1 Department of Ophthalmology, Tsukazaki Hospital, Himeji, Japan; 2 Department of Technology and Design Thinking for Medicine, Hiroshima University Graduate School, Hiroshima, Japan

## CLINICAL IMAGES

**Figure 1:**
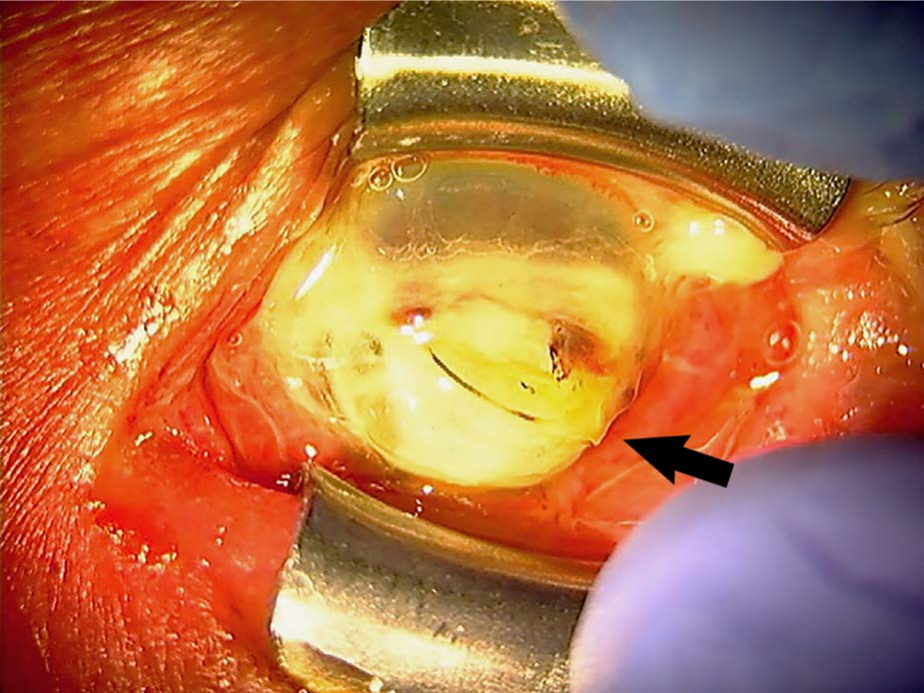
Slit-lamp photograph of the patient’s left eye at the time of his hospital visit. The lower part of the cornea is melted and the central part of the cornea is perforated, with an extruded intraocular lens and a prolapsed iris (indicated by the arrow).

A 79-year-old man receiving regular, once-monthly ophthalmologic conservative treatment for left chronic dacryocystitis had a normal cornea on 10 March 2020. On 5 April, he noticed decreasing left visual acuity. Using telephone consultation, the patient’s doctor explained to him that he should visit him on 7 April. However, owing to the fear of contracting coronavirus disease 2019, he cancelled his scheduled appointment on 7 April. On 11 May, he noticed significant left visual impairment with severe pain and promptly visited an ophthalmology clinic. As left corneal perforation was observed, he was urgently referred to our hospital. His left cornea had melted and was perforated from the centre to the lower part, exposing the intraocular lens and iris (Fig. 1). We made the working diagnosis of corneal melting and perforation secondary to chronic dacryocystitis. Methicillin-sensitive *Staphylococcus aureus* (MSSA) was detected in culture and sensitivity tests. Emergency evisceration was performed. Fluoroquinolone antibacterial eye drops, commonly used as antibacterial agents, have strong antibacterial activity against pathogenic bacteria causing various bacterial infections, including MSSA [1,2] Regardless of the underlying aetiology, breakdown of the corneal epithelium is the main pathologic mechanism of corneal ulceration. If the integrity of the corneal epithelium is compromised by any means, it can result in corneal ulcer and perforation [3]. If the patient had attended his scheduled appointment on 7 April after noticing decreasing left visual acuity and had received routine ophthalmologic examination and infection treatment with a sensitive drug, even if a corneal ulcer would have developed, it is highly possible that the serious complication of corneal perforation could have been prevented.

This case underscores the necessity of maintaining ophthalmologic consultations for patients with ocular morbidity experiencing ‘warning signs’, such as acute visual loss.

## ETHICAL APPROVAL

This case report was conducted according to the principles of the Declaration of Helsinki.

## CONSENT

Written informed consent was obtained from the patient.

## GUARANTOR

Daisuke Nagasato.
